# Industry Use of Evidence to Influence Alcohol Policy: A Case Study of Submissions to the 2008 Scottish Government Consultation

**DOI:** 10.1371/journal.pmed.1001431

**Published:** 2013-04-23

**Authors:** Jim McCambridge, Ben Hawkins, Chris Holden

**Affiliations:** 1 Faculty of Public Health & Policy, London School of Hygiene & Tropical Medicine, London, United Kingdom; 2 Department of Social Policy & Social Work, University of York, York, United Kingdom

## Abstract

Jim McCambridge and colleagues analyze industry submissions to a Scottish Government consultation on whole-population approaches to alcohol policy.

**Summary Points**

- We examine how research evidence is used in alcohol industry submissions made to a Scottish Government consultation in 2008 to advocate policies in line with their commercial interests.
- Industry actors consistently oppose the approaches found in research to be most likely to be effective at a population level without actually engaging with the research literature in any depth.
- Strong evidence is misrepresented and weak evidence is promoted. Unsubstantiated claims are made about the adverse effects of unfavoured policy proposals and advocacy of policies favoured by industry is not supported by the presentation of evidence.
- The potential for corporations with vested interests to interfere with the evaluation of scientific evidence by policy makers needs to be restricted for effective policies to be designed.
- Studies of the nature of alcohol industry and other corporate influences on public policies can be informed by work already conducted on the tobacco industry.

Like tobacco, alcohol is responsible for approximately 4% of the global burden of disease [1]. A range of policy options have developed in response to growing concerns about the scale of the problems caused by alcohol. In the international research literature there is broad consensus that measures to raise the price of alcohol and control its availability, along with restrictions on marketing activities, are the most effective measures [2]–[5]. These whole-population approaches involve reducing aggregate consumption at the population level. Conversely, there is little evidence for the effectiveness of some widely used approaches such as school-based education [5].

Alcohol industry actors, defined as all those involved with the production, supply, or sale of alcohol [6], do not have identical commercial interests and policy preferences [7]. They have consistently opposed whole-population approaches, however, favouring instead targeted interventions that focus on a supposedly problematic minority of drinkers and emphasising the role of individual responsibility [8],[9]. Industry actors have been strongly criticised for producing incomplete and distorted views of the evidence [10],[11] and influencing research funding and publications in biased ways [11]. These tactics mirror those of the tobacco industry [12], to which some producer organisations are closely related [13]. Unlike the tobacco industry, which has been excluded from direct influence in policy making in many countries, alcohol industry actors continue to exercise strong influence on alcohol policies across the world. For example, they have been implicated in the actual drafting of policy documents in low-income countries [14], and can lead public education in high-income countries [15],[16]. This discrepancy between the tobacco and alcohol industries has become more noteworthy as evidence on alcohol industry practices accumulates [17].

## Case Study

We examine formal submissions to the Scottish Government's 2008 consultation on “Changing Scotland's relationship with alcohol" [18]. This was the first governmental publication within the UK to adopt a whole population approach to alcohol policy, including measures to introduce minimum unit pricing (MUP; Text S1). MUP was championed by public health campaigners including the British Medical Association as an effective approach to tackling unacceptably high levels of alcohol problems, in line with the most recent WHO sponsored review of the policy options [5]. The election of a Scottish National Party (SNP) minority government in 2007 meant that for the first time since the establishment of the devolved Scottish Parliament in 1999, the Scottish Government was not formed by the main UK-wide political parties. The willingness of the SNP administration to explore innovative policy approaches and to engage with public health stakeholders created the conditions in which legislation introducing MUP was later passed [19]. It was subsequently accepted by the UK Government for England and Wales [20].

We retrieved 27 separate submissions by industry actors made during the public consultation process from the Scottish Government's website ([21]; a separate bibliography is presented in Text S2). We extracted and categorized the main evidential claims made and evaluated these claims against the most authoritative and up-to-date international review of the effectiveness of alcohol policy measures by Babor and colleagues [5]. We identify four main methods used by industry actors in linking evidence to policy, which are presented here according to our evaluation of their significance, defined primarily in terms of frequency and prominence across the documents as a whole. It is not being suggested that each method was used in every submission. In the text and boxes that follow, we provide illustrative examples, and interested readers are encouraged to access individual submissions directly, via the links provided in Text S2.

## Summary of the Industry Approach

The views articulated on the nature of the alcohol problem facing Scottish society and appropriate policy responses are consistent across submissions. This position is encapsulated in the Executive Summary produced by the Wine and Spirit Trade Association (WSTA) [22]. The WSTA claims to share the goal of the Scottish Government in reducing alcohol-related harm and seeks to work in partnership in developing an evidence-based approach to policy which can command high levels of public support. It is necessary, they argue, “to tackle the minority with alcohol problems – the drinkers rather than the drink" and “policies which punish all drinkers for the misconduct of a few" would be unfair [22].

Commitments to the use of evidence in policy making are often articulated. For example, the Portman Group (PG; an industry-wide social aspects and public relations organisation [11]) states that “the Scottish Government has both a duty and a right to help protect society against the adverse consequences of alcohol misuse, provided that it does this on the basis of the best available evidence and uses policy measures that are based as far as possible, on broad consensus within society [paragraph 2.6]…We believe in evidence-based policy making. If the evidence base is lacking, research should be undertaken rather than policy be introduced on a hunch" (4.11 in [23]).

### Misrepresentation of Strong Evidence

Industry submissions were frequently hostile to the whole-population approach advocated by the Scottish Government as well as to many, but not all, of the specific proposals made. Many submissions were critical of the evidence base underpinning the proposals, and based their opposition, at least in part, on evidential rather than on commercial grounds (see Box 1). These statements imply some evaluation of evidence, which is not presented in any of the submissions. Only in one instance was this omission declared: “We have not sought to carry out a detailed analysis of the evidence base referred to in the strategy in our response but we would observe that it is very selective" (WSTA page 8 in [22]). Industry actors thus accuse policy makers of possessing weak evidence for their policy proposals and selectivity in the presentation of evidence, though they neither evaluate the evidence themselves nor make use of evaluations by others.

**Box 1. Criticisms of the Evidence Underpinning the Scottish Government's Proposals**

Diageo [31]: “We think that the discussion paper lacks an overall framework for reducing alcohol-related harm and that many of the individual proposals are ill thought-out, are based on incomplete, selective and in some cases entirely absent evidence, and that they are likely not only to be ineffective in tackling minority alcohol misuse, but would also create unintended negative consequences" [page 4]…Diageo is concerned that the paper currently lacks evidence, or presents policy proposals based on evidence that is inadequate or patchy" [page 8].

Portman Group [23]: “Population-wide control policies, including restrictions on availability and price, are likely to be ineffective…[paragraph 4.5] Attempting to tackle problems through reducing per capita consumption (e.g. through taxation or restrictions on availability) is untargeted, unfair and likely to be ineffective. Indeed such an approach has been widely discredited in research studies" [paragraph 4.6].

SAB-Miller [26]: “It is predicated on the improbable assumption that raising the price of alcohol will make those who misuse alcohol behave differently" [paragraph 22].

Sainsbury's [30]: “We are concerned that the current proposals as they stand lack a strong evidence base and will result in a number of unintended consequences" [page 2].

Scotch Whisky Association [51]: “The consultation document fails to set out any firm evidence base on the relative merits and effectiveness of each of the measures being consulted to reduce alcohol misuse" [paragraph 2.9].

Scottish Beer and Pub Association [25]: “Where figures for the scale of the problem can only be described as “estimates" and are not “robust" we should be careful to place the appropriate level of emphasis on them…in respect of a number of pieces of research produced by the Scottish Government in the run up to the publication of its discussion paper we must question the basis on which some of this research has been produced" [page 12].

Tesco [52]: “The consultation makes many claims regarding the impact of price on consumption. Yet there is little in the way of evidence" [page 8].

Wine & Spirit Trade Association [22]: “The strategy is based on untested assumptions and a weak evidence base. There is no evidence that controlling access to alcohol across the board is the key to reducing consumption and alcohol-related harm and that reducing overall consumption will necessarily reduce harmful drinking" [page 2].

WM Morrison [28]: “Sadly, the actions proposed in the discussion paper do not follow from the evidence presented. Nor is there any evidence provided to back the assumption that by reducing overall consumption a reduction in harmful drinking will automatically follow" [paragraph 4.2].

According to the PG [23], “There is a raft of contradictory evidence of the influence of price and promotions on harm. In the absence of strong evidence, it seems imprudent to tackle alcohol misuse by acting against price and promotions" [paragraph 4.13]. No details of any contradictory evidence are provided here or in any of the other industry submissions and this statement conflicts with what is known in the scientific literature [5]. PG cite a seminal text elaborating the nature of the whole population approach [24], in support of their statement that this approach has been “widely discredited" [paragraph 4.6, see also Box 1]. Approximately half the submissions (n = 14) do not provide any references and all references provided in the other 13 submissions are provided in Text S3 [21].

There are repeated claims that advertising encourages only brand switching and does not lead to greater consumption, despite evidence to the contrary. The review of relevant research by Babor and colleagues indicates that marketing successfully recruits young people to drinking earlier than would be the case otherwise and increases consumption among existing drinkers [5]. According to the Scottish Beer and Pub Association (SBPA) [25]; “the overwhelming consensus of academic studies in this area concludes that it does not drive overall increases in category consumption in the total population" [page 6]. No references are provided. SAB-Miller [26] state that “There is, to our knowledge, no conclusive evidence that advertising causes underage drinking or alcohol abuse. There is evidence, however, that advertising bans or censorship have been ineffective" [paragraph 39]. To support this claim, an incomplete citation is provided to a small study of the effect of advertising on 166 fifth and eighth grade schoolchildren's alcohol expectancies ([27]; see also Text S3). Similarly, the large literature demonstrating the limited impact of education – leading Babor and colleagues to conclude that “education alone is too weak a strategy to counteract other forces that pervade the environment" [5] – is misrepresented. According to the WSTA [22], “Many commentators have attacked education as being ineffective in changing drinking behaviour. In fact there has been very little research in the area" [page 32].

### Promotion of Weak Evidence

Whilst industry actors misrepresent the international evidence [5] used by the Scottish Government, they draw on far weaker sources for their own positions. Their emphasis on public support makes opinion polls a key form of evidence. The SBPA [25], Morrisons [28], and ASDA [29] commissioned opinion polls themselves. ASDA [29] presents full results of a survey of 10,109 customers in the form of a tabulation of responses to four questions [page 3]. Morrisons [28] provides data on alcohol consumption at home, having reduced by 31% whilst consumption in other locations has increased by 14% in the 3-year period 2005–2008 (paragraph 5.3, page 6 in [28]). ASDA [29] similarly present their own market research which appears to show that under-age drinkers are half as likely to try to obtain alcohol from a large supermarket (39%) as they would from their parents (86%), an off-licence (78%) or a small convenience store (92%) (page 18 in [29]). Sophisticated internal industry data is not provided, though ASDA [29] and Sainsbury's [30] do both offer to share data with government.

Market research is also presented as intervention evaluation evidence. The outcome data in these reports are by their nature unable to provide evidence of changes in behaviour or reducing harms, as is implied. For example, referring to one of their campaigns, Diageo [31] suggest “Evaluation has shown the advertising to have a positive impact: more than 60% of those surveyed by the media evaluation agency Millward Brown said they were more likely to consider drinking responsibly following the adverts" [page 9]. Further claims are made about the effectiveness of industry initiatives. For example, Diageo [31] refer to a “successful trial" of a student unit awareness campaign in Glasgow, without providing any details of study design or criteria for success, and go on to cite data on the numbers later reached by this campaign as evidence of impact, rather than actual impact on behaviour [page 9]. Box 2 provides an example of a heavily promoted but weak intervention evaluation study [32]. Interestingly, the industry funded and extensively criticised International Centre for Alcohol Policies (ICAP) [10], a prolific producer of research reports, is only referred to by Pernod-Ricard and Diageo here.

**Box 2. A Model Intervention and Its Evaluation**

A community partnership in the small market town of St.Neots in Cambridgeshire was widely cited as a model initiative, for example being described as successful or effective by three of the four main supermarkets. The WSTA provided a detailed account of the initiative and evaluation data provided by the local police and trading standards officers. The project “aimed to improve recognition amongst enforcement authorities and the wider community that retailers are in fact often the victims of attempted under-age purchasing and should be seen as the front line of enforcement rather than the cause of the problem" [WSTA [22] pages 34-7]; see also Tesco [52] pages 4–6]. A 94% decrease in under-age people found in possession of alcohol described a change from 32 offences over the first nine enforcement operations to 1 offence in the 10^th^ and 2 in the 11^th^ operation (details of timing not provided). Pre–post comparison of 335 antisocial behaviour incidents in August to 196 in February yielded an overall reduction in antisocial behaviour incidents described as 42%. A decrease in alcohol-related litter at the skate park from 21 bottles and 86 cans on the first weekend of the project to 1 bottle and 8 cans on the final weekend produced a 92% decrease (the time periods differ from previously cited outcome measure).

The attention-grabbing percentage decreases are provided as evidence of benefits, and cited elsewhere, for example by SAB-Miller [paragraph 18 in [26]] without any alternative explanations for them being considered. All quantitative data produced in this evaluation study have been cited here and further information on this award winning and “ground breaking" [Tesco [52] page 6] project is available for inspection in the Retail of Alcohol Standards Group/WSTA publication on the county council website [32]. This report lacks any presentation of evaluation study methods and thus consideration of the possibility of biases. Claims of success involving quantitative data are made entirely on the basis of the before–after counts presented here, along with accounts of reductions in various problems without any quantification of them including a newspaper report that the local Member of Parliament receives fewer complaints about antisocial behaviour in one area. Other presentations of outcomes are that public perceptions and community confidence have been improved, without any information provided on how these data have been collected.

### Making Unsubstantiated Claims about the Adverse Effects of Policy Proposals

The submission documents contain a number of unsubstantiated claims about adverse, unintended consequences of the proposals. These claims are frequently repeated and are by their nature difficult to evaluate. Box 3 provides illustrations of the types of claims made. No evidential support for these claims is provided.

**Box 3. Claimed Adverse Effects of Policy Proposals**

ASDA [29]: “Believe that minimum pricing and a promotions ban will create incentives for the black market and criminals and illegal door to door sales" [page 10].

Sainsbury's [30]: Describe possible routes for cross-border shopping in England and Northern Ireland [paragraph 2.3].

Portman Group [23]: “Adopting a population-wide approach may not only fail to reduce misuse but it could perversely contribute to an increase in unhealthy drinking patterns and unregulated trading with the associated criminal activities [paragraph 4.8]…[increasing off-licence purchage age] could foster a feeling of resentment among young adults. It could also increase the appeal of alcohol to young people by creating a ‘mystique’ surrounding alcohol. Turning alcohol into a ‘forbidden fruit’ will only enhance its appeal to young adults looking to find ways of escaping the problems in their lives" [paragraph 5.28].

SBPA [25]: “May undermine the targeted initiatives, which may yield the highest results [sic] amongst the groups most heavily misusing alcohol" [page 6].

WSTA [22]: “We believe that it [increasing price] will create serious market distortions within the UK market…Additionally, this proposal risks increasing the sale of alcohol via unregulated “white van" type sales where the danger of sales to young people is high. We already see operators selling illicit alcohol and tobacco on to the poorer estates and we would expect this to become more prevalent" [page 19].

SAB-Miller [26]: “In countries where a minimum price has been introduced there have been unintended negative effects. By increasing the price of alcohol according to unit content the consequence is a shift from lower strength alcohol to those with higher alcohol content such as wines and spirits. This is especially problematic to harmful drinkers who are less responsive to changes in price and will continue to drink despite increased costs" [paragraph 26].

### Promoting Alternatives without Evidence

Industry actors present targeted harm reduction measures as the preferred alternative to the whole population approach. The latter is presented as being too simplistic and blunt an instrument. This is a false dichotomy, as these approaches are complementary and the actual evidence in favour of some targeted measures [5] is not presented. A sample of industry policy preferences is provided in Box 4. The submissions do not offer any evidence for this policy mix, and the underlying commercial interests of policy preferences [7] and conflicts of interests are consistently unacknowledged. Indeed, some industry actors claim their marketing activities contribute to harm reduction when they are directed towards increased sales and thus greater consumption. For example, Diageo [31] states that it “uses brand sponsorships to raise awareness of responsible drinking, including Guinness's sponsorship of the Rugby Premiership" [page 10].

**Box 4. Policy Preferences**

ASDA [29]: “We would urge policy-makers to concentrate on a combination of education, information and working with the on and off-trade to test out a wider range of voluntary measures to combat irresponsible drinking. Also, there is an urgent need to ensure that there is maximum enforcement of existing legislation" [page 7].

Diageo [31]: We strongly believe that a system of co-regulation is the most appropriate and effective approach to tackling alcohol misuse. Under co-regulation, the Government and the alcohol industry draw up standards together, which are strictly monitored and enforced, both within the industry and by Government through existing laws and regulations. But we also believe that individuals must take responsibility for their own actions, a principle which is unfortunately largely lacking in the discussion paper" [page 3].

Portman Group [23]: “An effective alcohol harm reduction strategy should cover a broad policy mix which comprises (a) effective enforcement of laws governing the sale and consumption; (b) self-regulation by those who produce, advertise and sell alcohol, and (c) encouragement of individuals to take personal responsibility for drinking choices" [paragraph 4.14].

SAB-Miller [26]: “There are less intrusive means by which the Scottish government can achieve its objectives than by imposing broad, population-based policies. These less intrusive means include education efforts on the laws against underage drinking, disorderly conduct, and sales to intoxicated or underage people, coupled with consistent and rigorous enforcement of these existing laws. Education and enforcement should be the cornerstone of Scotland's strategy to tackle alcohol misuse" [paragraphs 11 and 12].

Tesco [52]: “We consider that the Scottish Government's laudable objectives can best be achieved through a triple strategy of stricter enforcement of existing law, better education and effective partnership between those who sell alcohol, government, local authorities and enforcement authorities. Improved information and education about alcohol will help individuals make better decisions about their lifestyle choices and their alcohol consumption" [page 1].

## Discussion

The policy preferences of industry actors, with the interesting exception of greater law enforcement, are for policies such as industry self-regulation, public information, and education, which are the least likely to be effective [5]. Alcohol industry actors have been criticised previously for providing incomplete and distorted views of evidence [10],[11]. In this case study we demonstrate that industry actors ignored, misrepresented, and otherwise sought to undermine the content of the international evidence base on effective policies in order to influence policy. These tactics mean evidence-based policy-making is more difficult to achieve where industry actors are involved, in part by posing dilemmas for the research community about whether and in what circumstances to work with industry actors [33],[34].

There are few studies of alcohol industry documents in the public domain, with the exception of investigation of ICAP [10]. Wilkinson [35] examined five submissions to an Australian public consultation on new draft guidelines on low-risk drinking that were similarly critical of the research evidence. Miller and colleagues [36] analysed nine submissions concerning the industry-funded Drinkwise in a different Australian public consultation. In these cases, industry actors also made similar attempts to foster doubt about strong evidence and promote weak evidence, whilst appearing to be demonstrating corporate social responsibility [36]. Munro has examined a campaign against a tax increase on alcopops by the Distilled Spirits Industry Council of Australia, in which rhetorical commitments to the use of evidence for policy and selective release of industry data were also identified [37]. A crucial limitation of studies of documents designed for the public domain is that they tell us little about the less-visible means of industry influences on policy, nor about the success of these efforts [38]. We have used interviews with industry and other policy actors elsewhere to attempt to rectify this deficit [9], and these have begun to yield valuable insights into the role of lobbying in policy making in England and Scotland [19],[39].

Investigations of the strategies of alcohol industry actors may benefit from comparisons with other industries, and particularly with the tobacco industry. Access to internal tobacco industry documents offers researchers extensive knowledge of the political goals, strategies, and tactics of transnational tobacco companies [40]–[42]. Bero [43] provides a useful summary of the tactics used by tobacco companies in relation to evidence about the harms caused by tobacco. These include attempts to deceive the public and policy makers by hiding information held by companies and claiming that advertising and marketing are aimed only at persuading existing smokers to switch brands rather than also attracting new users, as claimed here.

In addition to co-ownership of alcohol and tobacco corporations (such as Miller and Phillip Morris [13],[44]), other possible means of sharing corporate experiences and tactics across industries include movements of senior personnel between industries and use of the same public relations firms. Bero [43] describes how tobacco industry lawyers edited scientific papers written by industry-funded scientists, including deleting acknowledgements of industry sponsorship [43]. Whether the alcohol industry may have subverted the evidence base in similar ways, and if so to what extent, is unknown [11].

The influence of corporate vested interests on the technical and scientific issues evaluated by policy makers now generates concern in areas including the environment, energy, biotechnology, pharmaceuticals, and defence [45]. An important tactic in contemporary corporate lobbying, pioneered by the tobacco industry, is the construction of doubt about the content of scientific evidence [43],[46],[47], and this may underlie the approach taken in these submissions. This has been shown to be influential on policy makers, particularly in the U.S., across a wide range of health and environmental concerns, as well on public opinion [46],[48]. Corporate influences are also discernible on the framing of issues for investigation by alcohol researchers [49], providing many targets for further study.

The preceding discussion raises questions of what is, and should be, the role of industry actors within the policy process [33],[34]. In Westminster, alcohol industry actors have cultivated long-term relationships with the main political parties [39] and an apparent consensus amongst these parties that they should have a wide-ranging role in alcohol policies [16],[20]. However, no clear boundaries have been established which facilitate the legitimate representation of commercial interests whilst protecting against conflicts of interest in evaluation of evidence. Despite the commitment to MUP, the most recently published Alcohol Strategy in England defines the alcohol problem in a way promoted by industry actors and reinforces the previous government's commitment to placing partnership with industry at the heart of policy making [16],[20],[33].

Policy making is not a purely rational process, informed only by evidence. It is by definition political and thus subject to a wide range of influences, and this complexity warrants dedicated investigations. However, we suggest that the public interest is not served by industry actors' involvement in the interpretation of research evidence. There is no obvious evidential contribution to be made by the corporate public relations specialists who engage with policy makers [50]. Commercial conflicts of interest should be made explicit and policy makers should treat industry actors' interpretation of research evidence with extreme caution. It is for public debate whether and to what extent the health of the population may be compromised by the commercial interests of industry, and whether the apparent economic contributions of the alcohol industry fully take into account the health and other social costs their activities incur. For policy makers, key questions concern how the pursuit of commercial interests may conflict with broader public interests and lead to the marginalisation of scientific evidence in decision-making.

## Supporting Information

Text S1 Complete list of Scottish Government consultation questions (annex h in [18]).
(DOCX)

Text S2 Twenty-seven alcohol industry submissions.
(DOCX)

Text S3 All sources cited in industry documents.
(DOCX)

## Abbreviations

ICAP: International Centre for Alcohol Policies
MUP: minimum unit pricing
PG: Portman Group
SBPA: Scottish Beer and Pub Association
SNP: Scottish National Party
WSTA: Wine and Spirit Trade Association
