# Supplementary material for: Industry Use of Evidence to Influence Alcohol Policy: A Case Study of Submissions to the 2008 Scottish Government Consultation
Source: PLoS Med. 2013 Apr 23;10(4):e1001431. doi: 10.1371/journal.pmed.1001431 (PMC3635861; doi:10.1371/journal.pmed.1001431)
Supplement: Text S1 — Complete list of Scottish Government consultation questions (annex h in [18]). (DOCX) [file pmed.1001431.s001.docx]

**Supporting Information Text S1:** **Complete List of Scottish Government Consultation Questions (annex h in [**[**18**](#_ENREF_18)**])**

**Irresponsible Promotions and Below-cost Selling**

We invite views on whether regulations should be made, under the Licensing (Scotland) Act 2005, to:

• put an end to off-sales premises supplying alcohol free of charge on the purchase of one or more of the product, or of any other product, whether alcohol or not.

• put an end to off-sales premises supplying alcohol at a reduced price on the purchase of one or more of the product, or of any other product, whether alcohol or not.

• prevent the sale of alcohol as a loss-leader.

**Minimum Retail Pricing**

We invite views on the proposed principles on which a minimum pricing scheme for alcohol products should be established.

**Information for Parents**

We will review current advice to parents and would welcome views on what particular information parents and carers would find helpful.

**Minimum Legal Purchase Age for Alcohol**

We invite views on whether we should raise the minimum age for off-sales purchases to 21 in Scotland.

**Social Responsibility Fee**

We invite views on the following aspects of a ‘social responsibility fee’:

• What criteria should be used to determine the types of premises (or specific premises) that should be subject to the fee? (e.g. late opening premises, or premises in a particular area) or conversely what criteria should be used to consider exemptions from the fee.

• How should the fee be determined? (e.g. based on rateable values, alcohol sales turnover)

• Should a fee be applied to Occasional Licences as well as Premises Licences?

• Should a similar fee be applied to other premises licensed under separate legislation? If so, what types of premises should be subject to a fee?

• Are there any other comments you would like to make on the operation of a social responsibility fee?

**Promotional Material in Licensed Premises**

We invite views on whether regulations should be made, under the Licensing (Scotland) Act 2005, to extend the existing regulations to:

• Prevent the display on licensed premises of promotional material relating to alcohol in a way visible to persons outside the premises.

• Prevent the use on licensed premises of any special display designed to promote sales of alcohol for consumption off the premises.

• Prevent on licensed premises any other promotional activity to induce the sale of alcohol for consumption off the premises.

**Separate Alcohol Checkouts**

We invite views on the desirability of creating separate checkouts for alcohol sales to help emphasise that alcohol is not an ordinary commodity. Also, the particular criteria that should be applied in determining which types of premises should be subject to any such arrangements; and whether there should be a requirement for alcohol checkout staff to be at least 18 years old.
