# Supplementary material for: Industry Use of Evidence to Influence Alcohol Policy: A Case Study of Submissions to the 2008 Scottish Government Consultation
Source: PLoS Med. 2013 Apr 23;10(4):e1001431. doi: 10.1371/journal.pmed.1001431 (PMC3635861; doi:10.1371/journal.pmed.1001431)
Supplement: Text S2 — Twenty-seven alcohol industry submissions. (DOCX) [file pmed.1001431.s002.docx]

**Supporting Information Text S2: 27 Alcohol Industry Submissions**

ASDA, *Common Ground with Fairness and Equity. ASDA’s Response to the Scottish Government’s Consultation Changing Scotland's Relationship with Alcohol*, 9th September 2008.

http://www.scotland.gov.uk/Publications/2008/11/26115423/320

Bacardi Brown Forman, *Response by Bacardi Brown-Forman Brands to the Scottish Government’s Discussion Paper Changing Scotland’s Relationship with Alcohol*, undated.

<http://www.scotland.gov.uk/Publications/2008/11/26115423/444>

Beam Global Spirits and Wine, Letter to the Scottish Government, 2 September 2008.

http://www.scotland.gov.uk/Publications/2008/11/26115423/85

BII [British Institute of Inn Keeping] Scotland, *Changing Scotland’s Relationship with Alcohol: A Discussion Paper on Our Strategic Approach. BII Scotland’s Response*, undated.

http://www.scotland.gov.uk/Publications/2008/11/26115423/337

The CEEV [Comité Européen des Enterprises Vins], *Consultation Questions; Consultation on Alcohol. Response from The CEEV* , via email, 29 September 2008.

http://www.scotland.gov.uk/Publications/2008/11/26115423/364

Chivas Brothers and Pernod Ricard, *Changing Scotland’s Relationship with Alcohol. A Joint Submission from Chivas Brothers Limited and Pernod Ricard UK Commenting on the Consultation Document from the Scottish Government*, 9 September 2008.

http://www.scotland.gov.uk/Publications/2008/11/26115423/458

The Cooperative Group, Letter to the Scottish Government, 8 September 2008.

http://www.scotland.gov.uk/Publications/2008/11/26115423/328

Diageo, *Changing Scotland’s Relationship with Alcohol. Response by Diageo to the Scottish Government’s Discussion Paper*, 3 September 2008.

http://www.scotland.gov.uk/Publications/2008/11/26115423/464

The Edrington Group Response, *The Scottish Government Strategy for Alcohol: The Edrington Group Response*, undated.

http://www.scotland.gov.uk/Publications/2008/11/26115423/454

The Gin and Vodka Association, *Consultation Questions; Consultation on Alcohol. Response from The Gin and Vodka Association*, via email, 30 September 2008.

http://www.scotland.gov.uk/Publications/2008/11/26115423/366

The National Association of Cider Makers, *Changing Scotland’s Relationship with Alcohol. A Discussion Paper on Our Strategic Approach. Response by The National Association of Cider Makers*, undated.

http://www.scotland.gov.uk/Publications/2008/11/26115423/323Noctis and SLNO [Scottish Late Night Operators Association], *Changing Scotland’s Relationship with Alcohol. A Response from by Noctis and SLNO*, 8 September 2008.

http://www.scotland.gov.uk/Publications/2008/11/26115423/326

The Portman Group, *Changing Scotland’s Relationship with Alcohol. A Response from The Portman Group*, September 2008.

http://www.scotland.gov.uk/Publications/2008/11/26115423/431

Punch Taverns, *Changing Scotland’s Relationship with Alcohol: A Discussion Paper on Our Strategic Approach. Response from Punch Taverns Plc*, undated.

http://www.scotland.gov.uk/Publications/2008/11/26115423/338

Retail of Alcohol Standards Group, *Response to Changing Scotland’s Relationship with Alcohol: A Discussion Paper on Our Strategic Approach*, undated.

http://www.scotland.gov.uk/Publications/2008/11/26115423/445

SAB-Miller, *Changing Scotland’s Relationship with Alcohol: A Discussion Paper on Our Strategic Approach. A Response by SAB-Miller*, undated.

http://www.scotland.gov.uk/Publications/2008/11/26115423/447

Sainsbury’s, *Sainsbury’s Submission to the Scottish Government’s Consultation Paper Changing Scotland’s Relationship with Alcohol: A Discussion Paper on Our Strategic Approach*, August 2008.

http://www.scotland.gov.uk/Publications/2008/11/26115423/448

The Scotch Whisky Association, *Response to the Scottish Government’s Consultation Paper Changing Scotland’s Relationship with Alcohol: A Discussion Paper on Our Strategic Approach*, undated.

http://www.scotland.gov.uk/Publications/2008/11/26115423/460

Scottish and Newcastle, *Changing Scotland’s Relationship with Alcohol. Scottish and Newcastle’s Response to the Consultation,* undated.

http://www.scotland.gov.uk/Publications/2008/11/26115423/451

The Scottish Beer and Pub Association, *The Scottish Beer and Pub Association’s Response to the Scottish Government’s Consultation Paper Changing Scotland’s Relationship with Alcohol: A Discussion Paper on Our Strategic Approach*, September 2008.

http://www.scotland.gov.uk/Publications/2008/11/26115423/405

The Scottish Grocers Federation, *Changing Scotland’s Relationship with Alcohol: A Discussion Paper on Our Strategic Approach. SGF Response*, undated.

http://www.scotland.gov.uk/Publications/2008/11/26115423/456

The Scottish Licensed Trade Association, *Response to the Scottish Government’s Consultation Paper Changing Scotland’s Relationship with Alcohol*, undated.

http://www.scotland.gov.uk/Publications/2008/11/26115423/457

The Scottish Retail Consortium Response, *Scottish Retail Consortium Response to the Scottish Government Consultation Changing Scotland’s Relationship with Alcohol: A Discussion Paper on Our Strategic Approach,* September 2008.

http://www.scotland.gov.uk/Publications/2008/11/26115423/331

Tesco, *Tesco Plc Submission to the Scottish Government Consultation Changing Scotland’s Relationship with Alcohol*, undated.

http://www.scotland.gov.uk/Publications/2008/11/26115423/462

William Grant and Sons, Letter to the Scottish Government, 9 September 2008.

http://www.scotland.gov.uk/Publications/2008/11/26115423/95

The Wine & Spirit Trade Association, *The Wine & Spirit Trade Association Submission to the Scottish Government Consultation Changing Scotland’s Relationship with Alcohol: A Discussion Paper on our Strategic Approach*, 4 September 2008.

http://www.scotland.gov.uk/Publications/2008/11/26115423/439

WM Morrison Supermarkets Plc, *Changing Scotland’s Relationship with Alcohol. Response to Discussion Paper*, undated.

http://www.scotland.gov.uk/Publications/2008/11/26115423/443
