# Supplementary material for: Industry Use of Evidence to Influence Alcohol Policy: A Case Study of Submissions to the 2008 Scottish Government Consultation
Source: PLoS Med. 2013 Apr 23;10(4):e1001431. doi: 10.1371/journal.pmed.1001431 (PMC3635861; doi:10.1371/journal.pmed.1001431)
Supplement: Text S3 — All sources cited in industry documents. (DOCX) [file pmed.1001431.s003.docx]

**Supporting Information Text S3: All sources cited in industry documents***

| ASDA | Hibell B *et al*., The ESPAD 2003 report: *Alcohol and other drug use in 35 European countries.*  ASDA / Serve Legal Research, March 2008, based on a survey of 205 Serve Legal visitors.  Institute of Alcohol Studies fact sheet – *Alcohol problems, causes and prevention*, December 2001.  Joseph Rowntree Foundation – *A minimum income standard for Britain*, July 2008.  *Review of the Social Responsibility Standards for the production and sale of Alcoholic Drinks*, published 22nd July 2008.  *Global Status Report: alcohol and young people*, David H Jernigan, 2001.  Reader’s Digest survey, April 2007, reported in *Daily Telegraph*, 18th April 2007.  Consultation paper, p.42.  Source: Institute of Alcohol Studies, drawing on statistics from *Eurobarometer 2002.* |
| --- | --- |
| Diageo | Swedish National Institute of Public Health, 2001, Where do we set the  limit? Sweden, 2004.  Drinking in Context: Patterns, Interventions and Partnerships, by Stimson, Grant, Choquet and Garrison (Routledge, 2007).  BMA Scotland written response to the Scottish Government’s  proposed strategy to tackle alcohol misuse in Scotland, August 2008). |
| NOCTIS | Source: CC Analysis  Source: CGA |
| Portman Group | *Smoking and drinking among adults 2006*, Office for National Statistics.  *Statistical Handbook 2007,* British Beer and Pub Association.  HM Revenue and Customs  Rose, G (1992) *The Strategy of preventive medicine*. Oxford University Press. Oxford.  Tuck, M (1980) *Alcoholism and Social Policy. Are we on the right lines?* Home Office Research Study No 65. HMSO. London.  Duffy, JC (1993) *Alcohol Consumption and Control Policy*. Journal of Royal Statistical Association. Series A (Statistics in Society), 156, (2).  *International Survey of Alcoholic Beverage taxation and Control Policies,* Brewers Association of Canada 1993.  *Review of the social responsibility standards for the production and sale of alcoholic drinks,* KPMG LLP, 11 April 2008.  Department for Transport data.  *Alcohol Awareness Week 2007 Evaluation Report,* Scotch Whisky Association.  *Setting the responsible drinking agenda*, The Portman Group, April 2008.  *Effects of beverage alcohol taxes and prices on consumption – a systematic review and meta-analysis of 1003 estimates from 112 studies*, Wagenaar, A. Salois, MJ. Komro, KA. Presented at the 34th Annual Alcohol Epidemiology Symposium of the Kettil Bruun Society for Social and Epidemiological research on Alcohol, Victoria, British Columbia, June 2-6, 2008.  *Help Note on multi-purchase, on-trade promotions*, Portman Group.  Furnham, A. (2002) *Growing up with Advertising*, The Social Affairs Unit.  Fisher, J (1993) *Advertising, alcohol consumption and abuse: a worldwide survey.* Westport Connecticut: Greenwood Press.    *Advertising and the misuse of alcohol*, FDS International and Volterra Consulting, June 2008.  *Review of the social responsibility standards for the production and sale of alcoholic drinks,* KPMG LLP, 11 April 2008.  *Setting the responsible drinking agenda*, The Portman Group, April 2008.  *Drinking: adults’ behaviour and knowledge in 2007*, Office for National Statisitics [sic] |
| SAB-Miller | US Federal Trade Commission, 2003.  Mallie J. Paschall, Joel W. Grube, Carol Black, and Christopher L. Ringwalt;  *Is Commercial Alcohol Availability Related to Adolescent Alcohol Sources and Alcohol Use? Findings from a Multi-Level Study; Journal of Adolescent Health*; 2007 August; 41(2): 168–174. [available at: <http://www.pubmedcentral.nih.gov/articlerender.fcgi?artid=2213632>].  Responsible Retailing Forum [available at: <http://www.rrforum.org>].  Cambridge News. *The party's over for underage drinkers*. 07/07/2007.  [available at: <http://www.cambridge-news.co.uk/cn_news_home/DisplayArticle.asp?id=328865>].  European Commission, Attitudes towards Alcohol, 2007, [available at:  http://ec.europa.eu/public_opinion/archives/ebs/ebs_272b_en.pdf].  Ryan, S., *Alcopop drinkers 'turning to spirits'*, The Australian, 29/05/08, [available at:  http://www.theaustralian.news.com.au/story/0,25197,23775465-5013871,00.html].  SABMiller plc; *Code of Commercial Communication***;** [available at:  <http://www.sabmiller.com/sabmiller.com/en_gb/Our+responsibility/Our+10+sustainable+development+priorities/Responsible+drinking/Code+of+Commercial+Communication.htm>].  Ipsitz,, A. Brake, G., Vincent. E., and Winters, M *Another Round for the Brewers: Television Ads and Children’s Alcohol Expectancies’, Northern Kentucky University.*  Grube, J. (2003). *Alcohol in the media: drinking portrayals, alcohol advertising, and alcohol consumption among Youth*, in National Academy of Sciences 2003 Report, pg. 613. |
| Sainsbury | A new way of tackling public under-age drinking’. Community Alcohol Partnerships. RASG and Cambridgeshire County Council).  KPMG Study |
| Scotch  Whisky  Association | Scottish Parliament written answer (S3W-11326) 17 April 2008.  Scotland’s Futures Forum. Approaches to Alcohol and Drugs in Scotland, A Question of Architecture (2008), Page 44.  61st World Health Assembly Paper WHA61.4, 24 May 2008.  Mäkelä, P., Bloomfield, K., Gustafsson, N.-K., Huhtanen, P., Room, R. (2007). Changes in volume of drinking after changes in alcohol taxes and travelers’ allowances: results from a panel study. *Addiction*, *103*, 181-191.  Scottish Government Spokesman 24 June 2008.  The Irish Competition Authority’s submission to the Government Alcohol Advisory Group (January 2008). The Scotch Whisky Association Response to the Scottish Government Alcohol Consultation Paper 28.  Mäkelä, P., Bloomfield, K., Gustafsson, N.-K., Huhtanen, P., Room, R. (2007). Changes in volume of drinking after changes in alcohol taxes and travelers’ allowances: results from a panel study. *Addiction*, *103*, 181-191; Manning, W., Blumberg, L., & Moulton  (1995). The demand for alcohol: the differential response to price. *Journal of Health Economics*, *14*, 123-148.  Grunewald, P.J., Ponicki, W.R., Holder, H., Romelsjö, A. (2006). Alcohol prices, beverage quality, and the demand for alcohol: quality substitutions and price elasticities. *Alcoholism:* *Clinical and Experimental Research*, *30*, 96-105; Österberg, E., & Karlsson, T., Eds. (2003). *Alcohol policies in EU member states and Norway: a collection of country reports*. Helsinki: National Research and Development Center for Welfare and Health (STAKES).  Irish Department of Health and Children: Teenagers’ views on solutions to alcohol misuse (April 2008, Page 22).  Johnston, L.D., O’Malley, P.M., Bachman, J.G., & J.E. Schulenberg (2007). *Monitoring the Future: National Results on Adolescent Drug Use*. Rockville, MD: US Department of Health and Social Services.  Wine and Spirits Daily (19 August 2008).  Scottish Parliament written answer (S3W-11326) 17 April 2008. Public Health Minister, Scottish Parliament Debate (30 June 2008).  Scottish Parliament written answer (S3W-11326) 17 April 2008. Public Health Minister, Scottish Parliament Debate (30 June 2008).  Hibell, B., Andersson, B., Bjarnason, T., Ahlström, S., Balakireva, O., Kokkevi, A., et al. (2004). *The ESPAD Report 2003: Alcohol and drug use among students in 35 European countries.* Stockholm: Swedish Council for Information on Alcohol and Other Drugs (CAN) and the Pompidou Group at the Council of Europe. |
| Scottish and Newcastle | *Statistical Handbook 2007*, British Beer and Pub Association.  HM Revenue and Customs.  Alavaikko, M., & Österberg, E. (2000). The influence of economic interests on alcohol control policy: a case study from Finland. *Addiction, 95* (Supplement 4), 1573-1582; Andreasson, S., Holder, H., Norström, T., Österberg, E., Rossow, I. (2006). Estimates of harm associated with changes in Swedish alcohol policy: results from past and present estimates.*Addiction*, *101*, 1096-1105; Mäkelä, P., Bloomfield, K., Gustafsson, N.-K., Huhtanen, P., Room, R. (2007). Changes in volume of drinking after changes in alcohol taxes and travelers’ allowances: results from a panel study. *Addiction*, *103*, 181-191.  Baker, T.K., Johnson, M.B., Voas, R.B., & Lange, J.E. (2000). Reduce youthful binge drinking: call an election in Mexico. *Journal of Safety Research*, *31*, 61-69; Lange, J.E., Lauer, E.M., & Voas, R.B. (1999). Survey of the San Diego-Tijuana cross-border bingeing: methods and analysis. *Evaluation Review, 23*, 378-398.  Hibell, B., Andersson, B., Bjarnason, T., Ahlström, S., Balakireva, O., Kokkevi, A., et al. (2004). *The ESPAD Report 2003: Alcohol and drug use among students in 35 European countries.* Stockholm: Swedish Council for Information on Alcohol and Other Drugs (CAN) and the Pompidou Group at the Council of Europe. |
| SBPA | Office of National Statistics figures  *Family Food – A report on the 2003-04 Expenditure and Food Survey, a National Statistics publication by DEFRA, August 2005.*  “*Costs of Alcohol Use and Misuse in Scotland*” Report published in May 2008.  Office of National Statistics (ONS) 2007 [referred to but not referenced].  Opinion survey of Scottish respondents conducted for the BBPA in July 2008.  Brand Index study by CGA Research. |
| Scottish Grocers Federation | Safe. Sensible. Social. The next steps in the National Alcohol Strategy, Department of Health, June 2007.  The Relationship between Off-sales and Problem Drinking in Scotland, Scottish Executive, June 18, 2007.  Data from Nielsen Retail Tracking of Off-Trade and On-Trade Copyright Nielson 2008.  Grunewald, P.J., Ponicki, W.R., Holder, H., Romelsjo, A (2006). Alcohol prices, beverage quality, and the demand for alcohol: quality substitutions and price elasticities. *Alcoholism: Clinical and Experimental research*, 30, 96-1005; Osterberg, E., & Karlsson, T., Eds. (2003). *Alcohol Policies in EU member states and Norway: a collection of* *country reports*. Helsinki: National research and Development Centre for Welfare and Health (STAKES).  This threshold size has a precedent in UK legislation in relation to Sunday trading in England and shelf-edge labelling requirements throughout the UK. |
| Scottish Retail Consortium | *Source: HMRC/Nielsen/BBPA*  *Gen. Household Survey, ONS, 2006* |
| Wine and Spirit  Trade  Association | Health & Wellbeing Profiles 2008 [referred to but not referenced].  Alcohol Control Database [referred to but not referenced].  TNS World Panel Survey [referred to but not referenced].  *Source: World Health Organisation, Regional Office for Europe, Alcohol Control Database.*  HMRC Consumption Data [referred to but not referenced].  ICM Research Poll [referred to but not referenced].  *Source: Monitoring the future: National Results of Adolescent Drug Use – US Dept of Social Services.*  Ofcom and the ASA, “Young People and Alcohol Advertising” 16/11/07 |
| WM Morrison | COM [European Commission](2006) 625 final, Paragraph 5.2.2  COM[European Commission] (2006) 625 final, Paragraph 6.2.1  TNS Worldpanel  Survey of 1,000 Morrisons Shoppers in Scotland conducted by Nunwood in August 2008  Institute of Alcohol Studies “Alcohol: tax, Price and Public Health” – Page 6. |

* All sources cited in the order and format they are presented without any corrections or other editing. Some documents also referred to specific data sources in the text but did not reference them. These are not included here.
